# Chronic mild stress exacerbates atrial fibrillation and neutrophil extracellular traps formation through S100A8/A9 signaling

**DOI:** 10.1038/s41392-025-02199-7

**Published:** 2025-04-04

**Authors:** Shan Meng, Tao Huang, Zijun Zhou, Liming Yu, Huishan Wang

**Affiliations:** State Key Laboratory of Frigid Zone Cardiovascular Disease, Department of Cardiovascular Surgery, General Hospital of Northern Theater Command, Shenyang, Liaoning P. R. China

**Keywords:** Cardiology, Cardiovascular diseases, Molecular medicine, Inflammation

**Dear Editor**,

Atrial fibrillation (AF) is the most prevalent form of cardiac dysrhythmia, characterized by an alarming surge in global incidence. Epidemiological and clinical evidence has unveiled the correlation between psychosocial factors (e.g., depression, negative affectivity, and job strain) and the incidence of AF.^1^ Notably, research has revealed that psychological stress triggers cardiovascular diseases through activation of hypothalamic-pituitary-adrenal (HPA) axis, and dysregulation of autonomic nervous system as well as immune system.^2^ Critically, chronic inflammation and dysregulation of immune cells emerge as the major biological consequences.^2^ However, our understanding of the precise mechanisms remains limited. A comprehensive investigation into the specific nature of atrial alterations in response to psychosocial stressors would offer valuable insights into our understanding of this disorder.

To examine the direct impact of chronic stress on the atria, we developed a well-established chronic mild stress (CMS) model by exposing C57BL/6 mice to a randomized sequence of six stressors either every two days (CMS1) or daily (CMS2) for a duration of 30 days.^3^ The detailed experimental procedures are provided in the supplementary materials. Depressive-like behaviors were validated by an exposure frequency-dependent reduction in open-arm exploration time in the elevated plus maze, along with marked increases in the immobility duration during the forced swimming and tail suspension tests. Meanwhile, a significant reduction in sucrose consumption revealed an obvious anhedonic behavior in CMS-challenged animals (data not shown). Critically, CMS induced an exposure frequency-dependent atrial enlargement (CON: 4.500 ± 0.224 mm^2^, CMS1: 5.200 ± 0.133 mm^2^, CMS2: 6.600 ± 0.163 mm^2^) and abnormal electrical conduction patterns, characterized by increased conduction inhomogeneity and reduced conduction velocity (Fig. 1a). These data were corroborated by the elevated AF inducibility in CMS2 group (Fig. 1a), indicating that CMS induced atrial structural and electrical remodeling, predisposing the animals to AF. To further explore the mechanisms, mRNA-seq was performed on atrial tissues from the CON and CMS2 groups. A total of 485 upregulated and 299 downregulated differentially expressed genes (DEGs, *P*-value < 0.05 and fold change > 2) were identified. Notably, within the upregulated DEGs, *S100a8* (logFC = 5.24, *p* = 7.94e-07) and *S100a9* (logFC = 3.88, *p* = 1.11e-04) ranked 5th and 12th, respectively, based on their log fold change (logFC) values. The western blotting data also revealed obviously enhanced atrial S100A8/A9 expressions within the CMS2 group (Fig. 1b, left). Additional GO analysis showed that the upregulated DEGs were significantly enriched in biological processes associated with inflammatory response, chemotaxis and migration of immune cells, particularly neutrophils (Fig. 1b, left). Indeed, compared to the CON group, the CMS2 group exhibited the highest fold increase in the average proportion of neutrophils (from 0.018 to 0.080) among 10 types of immune cells analyzed (Fig. 1b, right). Taken together, these data indicated the potential role of neutrophils-associated S100A8/A9 signaling in CMS-induced atrial remodeling.

**Fig. 1:**
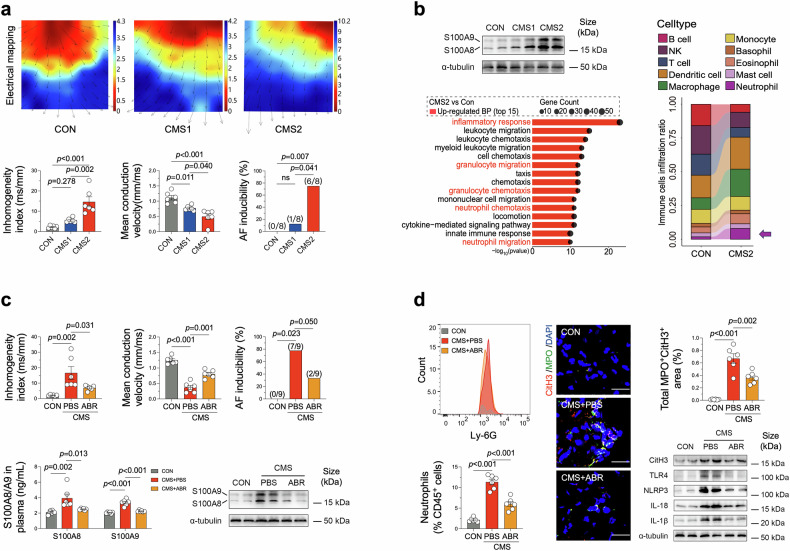
Atrial S100A8/A9 signaling in chronic mild stress-associated atrial fibrillation. **a upper** Left atrial electrical mapping obtained from Langendorff-perfused isolated hearts, with black arrows indicating the direction of electrical conduction on atrial surface. **a lower** Quantitative analysis on the inhomogeneity index and the mean conduction velocity calculated with computer algorithms (*n* = 6, one-way ANOVA). Quantitative analysis of AF inducibility (%). The successful AF induction was defined as the occurrence of AF in at least 2 out of 3 pacing episodes in each mouse (*n* = 8, Fisher exact test). **b left** Representative western blot images of S100A8/A9 in atrial tissue. The top 15 enriched Gene Ontology (GO)-Biological Process (BP) terms identified through analysis of the upregulated DEGs. **b right** A stacked bar chart illustrating the average ratio of immune cells (*n* = 6). **c upper** Quantitative analysis of the inhomogeneity index and mean conduction velocity (*n* = 6, one-way ANOVA). Percentage of successful AF induction (*n* = 9, Fisher exact test). **c lower** Quantitative analysis of S100A8/A9 expression in plasma and representative western blot images of S100A8/A9 in atrial tissue (*n* = 6, one-way ANOVA). **d left** Flow cytometry histogram of atrial tissue. Quantitative analysis of neutrophils (CD45^+^CD11b^+^Ly6G^+^) cells among immune cells (CD45^+^) in atrial tissue (*n* = 6, one-way ANOVA). **d middle** Representative immunofluorescence images depicting NETs in atrial tissues, identified using citrullinated histone H3 (CitH3, red), Myeloperoxidase (MPO, green), and DAPI (blue) staining. Quantitative analysis of NET-positive area (%) in atrial tissues (scale bar = 20 μm, *n* = 6, one-way ANOVA). **d right** Representative western blot images of CitH3, Toll-like receptor 4 (TLR4) and NOD-like receptor pyrin domain containing 3 (NLRP3) inflammatory pathway in the atria (*n* = *6*, one-way ANOVA). In this study, quantitative analysis of all Western blot and immunofluorescence images was conducted using Image J software

As pro-inflammatory alarmins abundantly expressed by neutrophils, S100A8/A9 contribute greatly to cardiac inflammation following myocardial infarction.^4^ However, the potential roles of S100A8/A9 in CMS-induced atrial damage remain unclear. Here, we employed a small-molecule inhibitor ABR-238901 (30 mg/kg, i.p., every 3 days) to block the activity of S100A8/A9 heterodimer complex in CMS model. Equal amount of PBS buffer was used as the vehicle. Interestingly, S100A8/A9 blockade inhibited atrial enlargement (CMS + PBS: 7.300 ± 0.300 mm^2^ vs. CMS + ABR: 6.200 ± 0.249 mm^2^, *p* = 0.010) and improved conduction patterns, thereby reducing the vulnerability to AF (Fig. 1c, upper). The administration of ABR-238901 showed no significant amelioration of depressive-like behaviors in CMS-exposed mice, as evidenced by behavioral testing (data not shown). However, compared to the CMS + PBS group, ABR-238901 administration successfully decreased plasma levels of S100A8 (3.942 ± 0.529 ng/mL vs. 2.501 ± 0.045 ng/mL, *p* = 0.013) and S100A9 (3.372 ± 0.168 ng/mL vs. 2.255 ± 0.040 ng/mL, *p* < 0.001), with comparable reductions observed in atrial tissue (Fig. 1c, lower). Furthermore, both flow cytometric (Fig. 1d, left) and immunofluorescence (data not shown) analyses revealed a significant enhancement of atrial neutrophil population. Notably, treatment with ABR-238901 effectively mitigated this elevation, leading to a substantial reduction in atrial neutrophil numbers (Fig. 1d, left). Similar decreases were also observed in peripheral blood samples (data not shown). Recently, S100A8/A9 have been recognized as the critical drivier of cardiac granulopoiesis and neutrophil Toll-like receptor 4 (TLR4)-NOD-like receptor pyrin domain containing 3 (NLRP3) activation.^4^ Additionally, the formation of neutrophil extracellular traps (NETs) has been implicated in AF pathogenesis.^5^ Here, we observed that CMS-treated murine atria exhibited markedly increased citrullinated histone H3 (CitH3), a marker of NETs, as well as the positive area of Myeloperoxidase (MPO) and CitH3 (Fig. 1d, middle). Furthermore, the downstream inflammatory mediators associated with S100A8/A9, including TLR4, NLRP3, IL-18, and IL-1β were also significantly enhanced in CMS-challenged atria (Fig. 1d, right). However, these effects were attenuated by ABR-238901, underscoring the critical role of S100A8/A9 in promoting CMS-induced atrial neutrophil activation and inflammation.

Our findings reveal a previously unrecognized role of S100A8/A9 signaling in CMS-induced atrial remodeling and arrhythmogenesis. Mechanistically, S100A8/A9 facilitates atrial inflammation, NET formation, and TLR4-NLRP3 activation, with transcriptome and cytometric data indicating a surge of atrial neutrophils in CMS-treated mice, potentially driven by neutrophil recruitment and granulopoiesis. While we acknowledge that neutrophil-specific gene deletion or bone marrow transplantation studies would provide more definitive evidence, our pharmacological experiments demonstrate that blocking S100A8/A9 mitigates neutrophil activation and atrial inflammation, highlighting their central role. Furthermore, CMS likely triggers neutrophil recruitment via stress-induced activation of the HPA axis and sympathetic nervous system, leading to increased granulopoiesis, cytokine release, and endothelial adhesion molecule expression, all of which create a pro-inflammatory environment. Elevated S100A8/A9 levels in CMS-challenged atria act as damage-associated molecular patterns, activating TLR4-NLRP3 signaling pathway to amplify inflammation and NET formation. Although the S100A8/A9 source organ in CMS is likely not limited to the atria, our findings establish S100A8/A9 as critical drivers of neutrophil-mediated atrial remodeling, providing a promising therapeutic target for psychological stress-associated atrial arrhythmia. Further studies are warranted to explore these mechanisms in greater depth.

## Supplementary information

Supplementary Material-SIGTRANS-15206R1

## Data Availability

All datasets generated for this study are included in the manuscript/Supplementary Files. The bulk RNA sequencing raw data presented in this research have been archived in the Genome Sequence Archive (Genomics, Proteomics & Bioinformatics 2021) in National Genomics Data Center (Nucleic Acids Res 2022), China National Center for Bioinformation/Beijing Institute of Genomics, Chinese Academy of Sciences (GSA: CRA019826). Additional supporting data for this study can be obtained from the corresponding author upon reasonable request.
