# Supplementary material for: Chronic mild stress exacerbates atrial fibrillation and neutrophil extracellular traps formation through S100A8/A9 signaling
Source: Signal Transduct Target Ther. 2025 Apr 4;10:108. doi: 10.1038/s41392-025-02199-7 (PMC11968817; doi:10.1038/s41392-025-02199-7)
Supplement: Supplementary file 1 — Supplementary Material-SIGTRANS-15206R1 [file 41392_2025_2199_MOESM1_ESM.docx]

Supplementary Materials for

**Chronic mild stress exacerbates atrial fibrillation and neutrophil extracellular traps formation through S100A8/A9 signaling**

Shan Meng^1^, Tao Huang^1^, Zijun Zhou^1^, Liming Yu^1^*, Huishan Wang^1^*

* Correspondence to: [huishanw@126.com](mailto:huishanw@126.com) or [lmyu2012@163.com](mailto:lmyu2012@163.com)

**This PDF file includes:**

Materials and Methods

Materials and Methods

Experimental animals

The experimental procedures were approved by the Animal Care Committee of the General Hospital of Northern Theatre Command (Approval No. 2023-51, 31 December 2023). Eight-week-old male wildtype C57BL/6J mice were obtained from HFK Bioscience Co., Ltd (Beijing, China) and housed under standard condition with ad libitum access to water and standard chow.

Chronic mild stress (CMS) procedures

The chronic mild stress (CMS) mouse model was developed with modifications based on previous studies ^1,2^. Mice in the stress groups were exposed to one randomly selected stressor either every two days (CMS1) or daily (CMS2) for a duration of 30 days, with a protocol ensuring that the same stressor was not repeated for three consecutive sessions to prevent adaptation. All CMS mice were subjected to stressors that were consistent in type, onset, and duration, which included: (1) inversion of light-dark cycle for 24 h, where mice were housed in a separate room with illumination from 8 pm to 8 am; (2) food deprivation for 24 h, where mice were deprived of food pellets; (3) squeezing for 4 h, where mice were squeezed within a transparent container (3×3×8 cm); (4) restriction for 4 h, where mice were restrained in an air-permeable 50mL conical tube; (5) horizontal oscillation for 2 h, where mice were subjected to oscillation using a shaking table (80 rpm); and (6) damp bedding for 12 h, where mice were placed in a cage with damp bedding and water. A separate group of animals raised under standard conditions served as the control group.

Elevated Plus Maze

The Elevated Plus Maze was positioned 50 cm above the floor and consisted of two open and two closed arms ^3^. Mice were introduced to the central zone for a 6-minute exploration period, and the time spent in the open arms was recorded using a camera and specialized software.

Forced swimming test

The forced swimming test was employed to evaluate depression-like behaviors in mice ^4^. Each mouse was placed in a glass cylinder (20 cm × 40 cm) filled with 30 cm of water at 25°C. The 6-minute test focused on observing immobility during the final 5 minutes, defined as passive upright floating without struggling.

Tail suspension test

Each mouse was suspended by its tail with adhesive tape, positioning them 50 cm above the ground in a head-down orientation ^5^. Immobility duration was defined as the passive hanging of the mouse with no observable movement within 5 minutes.

Sucrose preference test

The sucrose preference test assessed anhedonia by measuring preference for a 1% sucrose solution in a two-bottle-choice test after a 24-hour deprivation of food and water ^6^. The sucrose preference index (SPI) was calculated as: SPI = [sucrose water intake / (sucrose water intake + pure water intake)] × 100%.

Echocardiography

Atrial and ventricular function were measured with the ultrasound imaging system (D700, Vinno Technology Co., Ltd., Suzhou, China) as previously described a blinded manner ^7^.

Langendorff-perfused isolated hearts and electrophysiological mapping

Following euthanasia, the mice underwent thoracotomy, and their hearts were perfused with Tyrode's solution on a Langendorff-perfusion system ^7^. The 64-channel MEA system (MappingLab Ltd., Oxford, UK) was vertically contacted to the left atrium and mapping data were analyzed using EMapScope 5.0 software.

Atrial fibrillation (AF) induction and ECG recording

AF was induced by inserting an electrode catheter (Transonic Scisense Inc., Ontario, Canada) into the right jugular vein ^7^. Electrical stimulation was performed at 5 volts amplitude, 40 ms cycle length, 5 ms pulse width, and 10 pulse counts. AF was defined as a rapid and irregular atrial rhythm lasting at least 1 second ^8^.

Bulk RNA sequencing and data analysis

Total RNA was extracted from the atrial samples and sequenced using the Illumina NovaSeq X system in PE150 mode (*n=6* per group). The data were processed in R software (RStudio version 2023.06.0 Build 421) and the differentially expressed genes (DEGs) were identified with the "limma" package by applying a threshold of an absolute Log2 fold change (Log2FC) value greater than 0.263 and a *p*-value below 0.05. The heatmap was generated using TBtools-II software. Gene ontology (GO) enrichment analysis was performed through the DAVID platform (v3.5.20240101). The ratio of immune cells in each sample was analyzed using the "ImmuCellAI-mouse" package ^9^.

Western blot analysis

Western blot analysis was performed as described previously ^7^. Briefly, primary antibodies against S100A8 + S100A9 (ab288715, 1:1000, Abcam, USA), α-tubulin (12351s, 1:1000, Cell Signaling Technology, USA), Cit-H3 (ab281584, 1:1000, Abcam, USA), TLR4 (ab22048, 1:1000, Abcam, USA), NLRP3 (ab263899, 1:1000, Abcam, USA), IL-18 (57058, 1:1000, Cell Signaling Technology, USA) and IL-1β (sc-12742, 1:500, Santa Cruz, USA) were employed. Protein bands were detected with a Tanon image analyzer (Shanghai, China).

Immunofluorescent staining assay

Immunofluorescent staining assay was executed as detailed in prior studies^7^. Briefly, primary antibodies against S100A8 + S100A9 (ab288715, 1:200, Abcam, USA), Ly-6G (#88876, 1:200, Cell Signaling Technology, USA), Cit-H3 (ab281584, 1:200, Abcam, USA), Myeloperoxidase (ab300650, 1:200, Abcam, USA) were employed. The sections were viewed through a Nikon C2 Plus confocal microscope (Nikon, Tokyo, Japan), and the analysis of fluorescence quantification results was conducted using Image J software.

Flow cytometry

Atrial tissue was minced and incubated for 3 hours in a pre-warmed enzymatic digestion medium containing 0.1 g/mL collagenase II ([1148090](https://www.sigmaaldrich.cn/CN/zh/product/usp/1148090), Sigma-Aldrich, USA), 20 μg/mL DNase I (11284932001, Sigma-Aldrich, USA) and 5mM MgCl₂. Cell suspension was filtered through a 70 μm cell filter, purified with 2% BSA/PBS and then incubated with FITC anti-mouse CD45 (103108, Biolegend, 1:200), BV421 anti-mouse/human CD11b (101235, Biolegend, 1:200), BV510 anti-mouse Ly6G (127633, Biolegend, 1:200) and 7-AAD anti-mouse 7-AAD (00-6993-50, eBiosciences, 1:200) at room temperature in darkness for 15 minutes before analysis using FACS Celesta (BD Bioscience, USA). Once the doublets (by FSC and SSC) and dead cells (by 7-AAD^+^) were excluded, neutrophils were identified as CD45^+^, CD11b^+^, Ly6G^+^. Cells data analysis was performed using Flowjo software (TreeStar, USA).

Statistical analysis

Data were presented as means ± SEM (Standard Error of Mean). The student’s t-test was employed for analyzing two groups, while one-way ANOVA with Tukey multiple comparisons was utilized for analyzing multiple groups. The inducibility of atrial fibrillation (AF) was assessed using the Fisher exact test. The data were analyzed and visualized using GraphPad Prism 9 software (Version 9.0, San Diego, CA, USA).

**References**

1 Heidt, T. *et al.* Chronic variable stress activates hematopoietic stem cells. *Nat Med.* **20**, 754-758, (2014).

2 Schneider, K. M. *et al.* The enteric nervous system relays psychological stress to intestinal inflammation. *Cell*. **186**, 2823-2838.e2820, (2023).

3 Chen, B. *et al.* Hypocretin-1/Hypocretin Receptor 1 Regulates Neuroplasticity and Cognitive Function through Hippocampal Lactate Homeostasis in Depressed Model. *Adv Sci (Weinh)*, e2405354, (2024).

4 Ma, S. *et al.* Sustained antidepressant effect of ketamine through NMDAR trapping in the LHb. *Nature*. **622**, 802-809, (2023).

5 Cryan, J. F., Mombereau, C. & Vassout, A. The tail suspension test as a model for assessing antidepressant activity: review of pharmacological and genetic studies in mice. *Neurosci Biobehav Rev*. **29**, 571-625, (2005).

6 Liu, M. Y. *et al.* Sucrose preference test for measurement of stress-induced anhedonia in mice. *Nat Protoc*. **13**, 1686-1698, (2018).

7 Zhao, J. *et al.* Diminished α7 nicotinic acetylcholine receptor (α7nAChR) rescues amyloid-β induced atrial remodeling by oxi-CaMKII/MAPK/AP-1 axis-mediated mitochondrial oxidative stress. *Redox Biol*. **59**, 102594, (2023).

8 Yu, L. M. *et al.* Activation of PKG-CREB-KLF15 by melatonin attenuates Angiotensin II-induced vulnerability to atrial fibrillation via enhancing branched-chain amino acids catabolism. *Free Radic Biol Med*. **178**, 202-214, (2022).

9 Miao, Y. R. *et al.* ImmuCellAI-mouse: a tool for comprehensive prediction of mouse immune cell abundance and immune microenvironment depiction. *Bioinformatics*. **38**, 785-791, (2022).
